# Integrated multi-omics analysis reveals a pH-driven metabolic and translational switch in *Ureaplasma parvum*

**DOI:** 10.1128/spectrum.03463-25

**Published:** 2026-07-10

**Authors:** Hiroaki Hase, Yukiko Nakura, Yuki Shimada, Atsuko Nishino, Michiko Kodama, Itaru Yanagihara

**Affiliations:** 1 Laboratory of Stem Cell Regeneration and Adaptation, Graduate School of Pharmaceutical Sciences, The University of Osaka 13013, Suita, Osaka, Japan; 2 Department of Developmental Medicine, Research Institute, Osaka Women’s and Children’s Hospital, Izumi, Osaka, Japan; 3 Department of Obstetrics and Gynecology, Graduate School of Medicine, The University of Osaka 13013, Suita, Osaka, Japan; Oklahoma State University, Stillwater, Oklahoma, USA

**Keywords:** *Ureaplasma parvum*, proteomics, metabolomics, RNA modification, pH stress, multi-omics

## Abstract

Human ureaplasmas are minimal-genome bacteria and pathobionts of the urogenital tract. They must adapt to fluctuating pH conditions despite the absence of canonical transcriptional regulatory systems. However, the mechanisms underlying these responses remain unclear. This study aimed to construct a system-level model of pH adaptation in this minimal pathogen. We used an integrated multi-omics platform combining proteomics, metabolomics, and RNA modification profiling to construct a system-level model of pH adaptation. The results revealed a bifurcated strategy governed by the differential activation of preexisting, co-regulated functional modules. Under neutral pH conditions (pH 7), *Ureaplasma parvum* activated energy metabolism and upregulated ATP synthesis while forming a stress-counteracting proteostasis pathway. This may suggest a biological energy state under high stress conditions. Conversely, under acidic stress (pH 5), it activated biosynthesis/translation, showing significant upregulation of ribosomal proteins and accumulation of translation precursors and the polyamine spermidine. This may represent a state of expanded translational capacity. This adaptive switch is accompanied by dynamic reorganization of the epitranscriptome, highlighting the importance of post-transcriptional regulation. This study suggests mechanisms by which minimal organisms achieve adaptive plasticity through sophisticated post-transcriptional and metabolic control, providing a new framework for understanding *Ureaplasma* physiology and the biology of genome-reduced organisms.

**IMPORTANCE:**

Minimal bacteria challenge canonical views of cellular regulation. In organisms with radically reduced genomes and sparse transcription factors, how adaptive plasticity is achieved remains a core question. Our study proposes a model in which a simple physicochemical cue—extracellular pH—selects among prewired cellular programs, while post-transcriptional and epitranscriptomic layers fine-tune execution. The findings of this study suggest a multi-omics scheme for how organisms adapt to environmental changes and ensure survival without inducing new circuits or complex transcriptional regulation. Conceptually, it proposes regulation via RNA modifications in processes, such as metabolism, proteostasis, and translation. This framework may be generalizable to other genome-reduced microorganisms. Beyond microbiology, it provides design principles for synthetic biology and offers a mechanistic interpretation of phenotypic tolerance to stress factors. It may encourage the use of pH-linked epitranscriptome signals as measurable indicators of cellular state.

## INTRODUCTION

*Ureaplasma* species, members of the class Mollicutes, are among the smallest self-replicating pathogenic organisms. They are characterized by the absence of cell walls (1). One characteristic of *Ureaplasma* is the absence of a cell wall, which inherently confers resistance to beta-lactam antibiotics. To compensate for this lack of a cell wall, *Ureaplasma* incorporates exogenous cholesterol from the host environment into its cytoplasmic membrane, thereby maintaining membrane stability and structural integrity. They are common extracellular residents of the human urogenital tract, where they exist as pathobionts, commensals that can become pathogenic under certain conditions (2–4). Clinically, *Ureaplasma* has been implicated in various adverse health outcomes, including bacterial vaginosis (BV), preterm birth, and infertility. Its ability to thrive in the fluctuating chemical environment of the urogenital tract, particularly with respect to pH, suggests the presence of sophisticated adaptation mechanisms in this bacterium.

The physiology of *Ureaplasma* is governed by its unique energy metabolism. It has a degenerated glycolytic pathway and relies almost entirely on urea hydrolysis for ATP synthesis (5). This reaction, catalyzed by nickel-dependent urease (6), generates ammonia and increases the local pH. This creates a feedback loop in which the core metabolic processes of the organism actively remodel the environment, which is a key factor in both survival and pathogenesis (7). Although the central role of urease is well established, little is known about the broader, system-wide molecular adjustments that *Ureaplasma* uses to adapt to different pH conditions. This organism must navigate environments ranging from normal acidic vaginal conditions (pH ~4.5) to the more neutral pH of the upper reproductive tract or BV, with optimal growth occurring near pH 6. Therefore, understanding this response is crucial (8).

BV is the most common cause of vaginitis. According to the Amsel Criteria, a vaginal pH >4.5 is a diagnostic indicator of BV (9). In most bacteria, adaptation to pH stress is governed by complex transcriptional regulatory networks, often involving two-component systems and alternative sigma factors that modulate gene expression (10). However, *Ureaplasma parvum*, with a genome of only ~0.8 Mbp and <600 protein-coding genes, lacks many canonical regulatory systems (11). This “genomic paradox” raises a fundamental question: how does a minimal organism coordinate a robust and integrated response to environmental challenges such as pH stress?

Traditional reductionist approaches that focus on one gene or protein at a time are often insufficient to elucidate the complex system-level adaptations of such organisms. Multi-omics methodologies that integrate data from distinct molecular layers, including genomics, transcriptomics, proteomics, and metabolomics, offer a holistic view of cellular functions (12). By simultaneously capturing changes in proteins, metabolites, and other molecular components, these approaches can reveal emergent properties and regulatory mechanisms that would remain hidden when analyzing a single data type. In minimal organisms, where extensive transcriptional regulation is absent, it is reasonable to hypothesize that post-transcriptional, translational, and metabolic regulation play outsized roles, making a multi-omics strategy especially compelling (13, 14).

In this study, we used a comprehensive analytical platform that integrates proteomics, metabolomics, and RNA modification profiling to elucidate the mechanisms by which *U. parvum* adapts to different pH conditions (pH 5, 6, and 7). Our aim was to construct a system-level model of pH adaptation in this minimal pathogen. The results revealed a sophisticated, multilayered regulatory strategy: under pH 5 conditions, the organism enters a growth-quiescent preparatory state, whereas under pH 7 conditions, it shifts to a high-stress, survival-oriented state, characterized by enhanced energy production and defense against self-generated ammonia. This study demonstrates the remarkable adaptive capacity of *Ureaplasma* and establishes multi-omics as a powerful framework for deciphering the biology of microorganisms with highly reduced genomes.

## RESULTS

### Proteomic analysis reveals distinct, pH-dependent states

**Fig 1:**
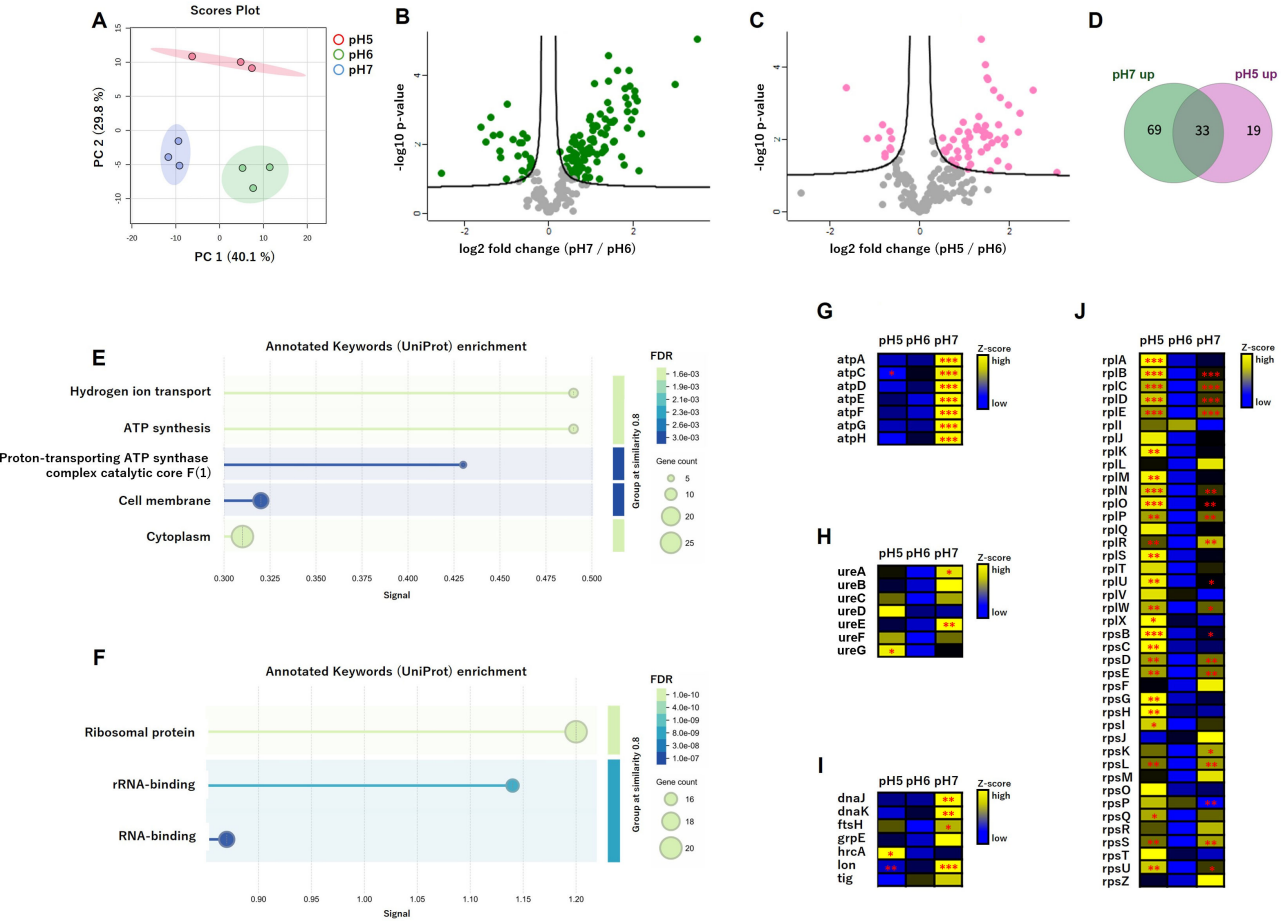
Proteomic analysis reveals distinct pH-dependent cellular states in *Ureaplasma*. (**A**) Principal component analysis (PCA) of proteomic data from *Ureaplasma* cultured at pH 5 (red), 6 (green), and 7 (blue). Each point represents one of three biological replicates (*n* = 3), showing clear clustering by pH. (**B and C**) Volcano plots showing the differentially abundant proteins. The horizontal axis represents the log2 fold change, and the vertical axis represents the −log10 *P* value. Panel **B** compares pH 7 with pH 6, and panel **C** compares pH 6 with pH 5. The black lines indicate the significance threshold (FDR < 0.05 and S0 = 0.1). Proteins outside these lines are considered significantly differentially abundant. (**D**) Venn diagram illustrating the overlap of proteins significantly upregulated at pH 5 and pH 7, both relative to pH 6. (**E and F**) Functional enrichment analysis of proteins upregulated at pH 5 (**E**) and pH 7 (**F**). The horizontal axis “Signal” indicates enrichment intensity relative to the genomic background using UniProt keywords, while dot size and color represent the number of associated proteins and the FDR (false discovery rate), respectively. Enriched terms were grouped at a similarity threshold of 0.8 (Jaccard index) to reduce redundancy; only the most representative term per group is shown. (**G–J**) Heatmaps showing the relative abundance (*z*-score) of specific protein families across all replicates: (**G**) F-type ATP synthase subunits, (**H**) urease subunits, (**I**) stress response proteins (chaperones and proteases), and (**J**) ribosomal proteins. Statistical significance was determined by Dunnett’s test using pH 6 as the control group. Asterisks indicate significant differences compared with pH 6 (*P < 0.05, **P < 0.01, ***P < 0.001).

To examine the effect of pH on *U. parvum*, cultures were prepared under three initial pH conditions, pH 5, 6, and 7, for multi-omics analysis. To assess the physiological state of the cultures at the 16-h sampling point, viable cell counts and medium pH were evaluated under the same initial pH conditions. Viable cells were detected under all three conditions at the sampling point, and the medium pH remained broadly consistent with each initial pH condition during this period. Additional time-course turbidity and pH measurements in scaled-up 20-mL cultures showed condition-dependent changes in relative culture turbidity and medium pH during prolonged culture, rather than a canonical logistic growth profile with a stable plateau (Fig. S1A through C). Quantitative proteomic analysis was performed on *Ureaplasma* cultured at pH 5, 6, and 7. Principal component analysis (PCA) of the resulting data demonstrated distinct clustering of the three pH groups, indicating a global and reproducible shift in the cellular proteome in response to nonoptimal pH (Fig. 1A). To identify the specific proteins driving this separation, we performed a differential abundance analysis. Volcano plot analysis revealed 102 significantly upregulated proteins at pH 7 (Fig. 1B) and 52 at pH 5 (Fig. 1C) under both stress conditions. A Venn diagram further illustrated that 19 of these proteins were uniquely upregulated at pH 5 and 69 were unique to pH 7, with 33 proteins being commonly upregulated under both stress conditions relative to the alternative pH (Fig. 1D).

Functional enrichment analysis of the proteins upregulated at pH 7, using UniProt keywords to concisely capture functional and localization properties, revealed a strong association with energy metabolism, with “ATP synthesis” and “Hydrogen ion transport” being the most significantly enriched categories (Fig. 1E). In contrast, the proteins upregulated at pH 5 were overwhelmingly associated with translation, showing a significant overrepresentation of the UniProt keywords “Ribosomal protein” and “rRNA-binding” (Fig. 1F). These functional enrichments were visually confirmed using heatmaps of specific protein families. At pH 7, multiple subunits of the F-type ATP synthase complex were markedly and consistently more abundant than those at pH 5 and pH 6 (Fig. 1G). The amount of urease subunits exhibited more diverse patterns, with some subunits showing an increasing trend at pH 5 or pH 7 compared with the optimal pH 6 (Fig. 1H). Of note, these data reflect protein abundance and not enzymatic activity. Furthermore, the pH 7 state was accompanied by a pronounced stress response, as evidenced by the significant and specific increase in the abundance of DnaK, DnaJ, and ATP-dependent Lon protease (Fig. 1I). Conversely, a heatmap of ribosomal proteins showed a coordinated and marked increase in the abundance of most ribosomal subunits at pH 5 compared with the other conditions (Fig. 1J).

### Metabolomic profiling corroborates the proteomic findings

**Fig 2:**
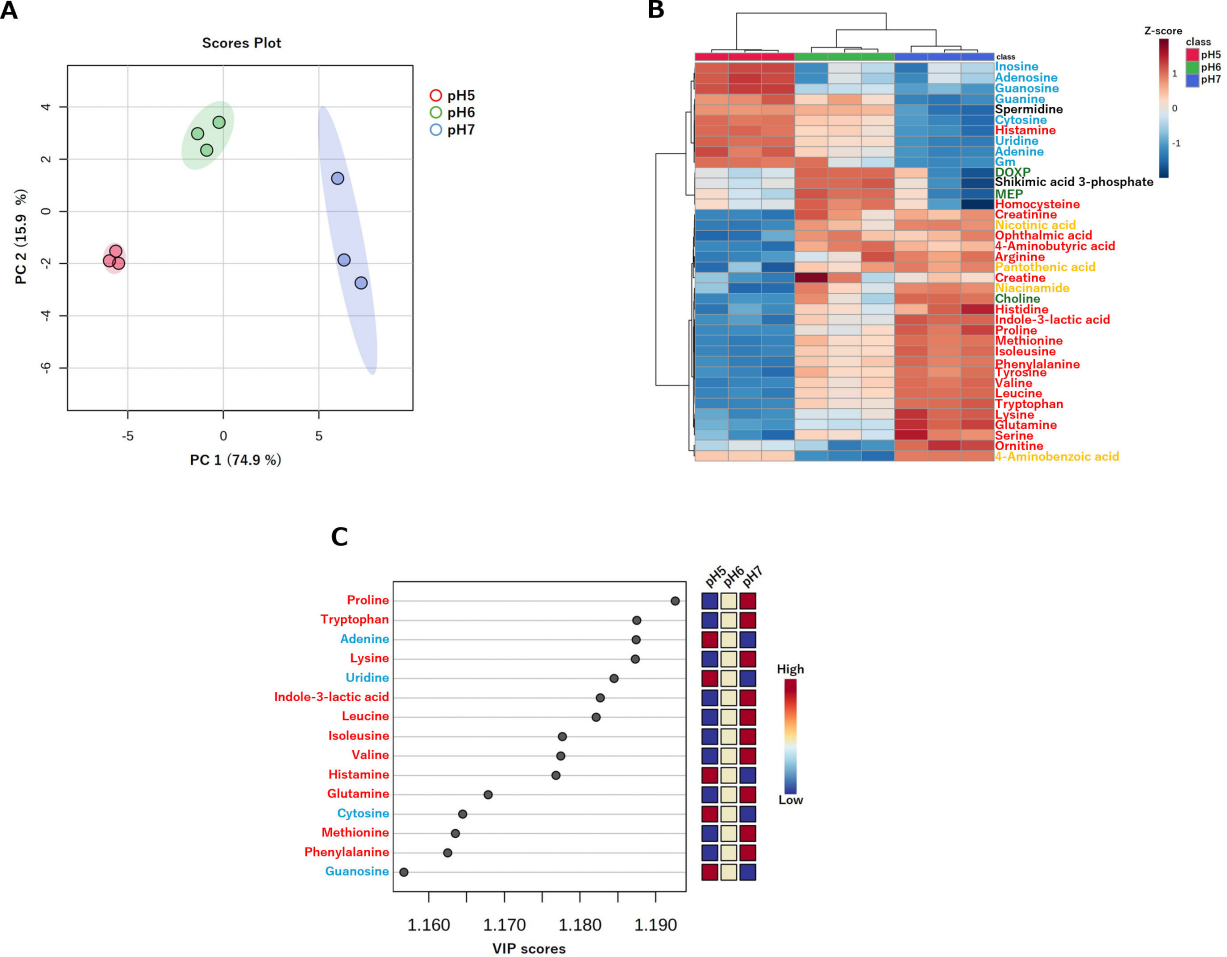
Metabolic profiles of *Ureaplasma* are remodeled in response to pH. All data are from *n* = 3 biological replicates per condition. (**A**) Unsupervised PCA plot of metabolomic data showing distinct separation of samples cultured at pH 5 (red), pH 6 (green), and pH 7 (blue). (**B**) Hierarchical clustering heatmap of 38 metabolites selected by one-way ANOVA with Tukey’s post-hoc test (FDR-adjusted *P* < 0.05), illustrating distinct metabolic signatures for each pH condition. Red indicates a higher relative abundance, and blue indicates a lower relative abundance. (**C**) Variable importance in projection (VIP) scores plot from the partial least squares discriminant analysis (PLS-DA), identifying the top 15 metabolites that contribute most significantly to the separation between pH conditions. A VIP score ≥1.0 is generally considered indicative of a meaningful contribution to the model. To facilitate interpretation, metabolites are color-coded and organized by pathway: amino acids and stress response (e.g., leucine, GABA, optamic acid; red text), nucleoside and nitrogen metabolism (e.g., adenosine, inosine, allantoin; blue text), vitamins and cofactors (e.g., nicotinic acid, PABA, pantothenic acid; yellow text), energy/membrane metabolism (e.g., lactic acid, DOXP, choline; green text), and others (black text).

We performed a metabolomic analysis to assess whether the observed proteomic shifts were accompanied by corresponding changes in metabolism. An unsupervised PCA plot showed a clear separation of the three pH groups, mirroring the proteomic results (Fig. 2A). Pairwise volcano plot analyses using pH 6 as the control condition further visualized the magnitude and significance of individual metabolite changes at pH 5 and pH 7, respectively (Fig. S2A and B). Hierarchical clustering of highly variable metabolites further demonstrated differences in metabolic profiles between conditions, revealing a clear boundary between the pH 5 and 6 groups and the pH 7 group (Fig. 2B). Contributing features to this clustering at pH 5 were nucleosides and nucleobases, including inosine, adenosine, guanosine, cytidine, and uridine, suggesting altered nucleotide metabolism under acidic conditions. Conversely, pH 7 showed relative accumulation of numerous amino acids. This separation was confirmed using a partial least squares discriminant analysis (PLS-DA) plot, a supervised analysis method (Fig. S2C). The variable importance in projection (VIP) score plot from the PLS-DA identified key metabolites contributing to this discrimination, confirming that amino acids were characteristic of pH 7, as suggested by the hierarchical clustering. Notably, the accumulated amino acids spanned diverse structural and functional classes, including the branched-chain amino acids (BCAAs) leucine, isoleucine, and valine; the aromatic amino acid tryptophan and its metabolite indole-3-lactic acid; the basic amino acid lysine; and the neutral amino acids glutamine and methionine, suggesting a broad rather than pathway-specific response. In contrast, the metabolic profile at pH 5 was characterized by the relative accumulation of adenine, uridine, cytosine, and guanosine, indicating that nucleotide metabolism was more prominently altered at pH 5 compared with pH 7 (Fig. 2C).

### Epitranscriptome is dynamically remodeled in response to pH

**Fig 3:**
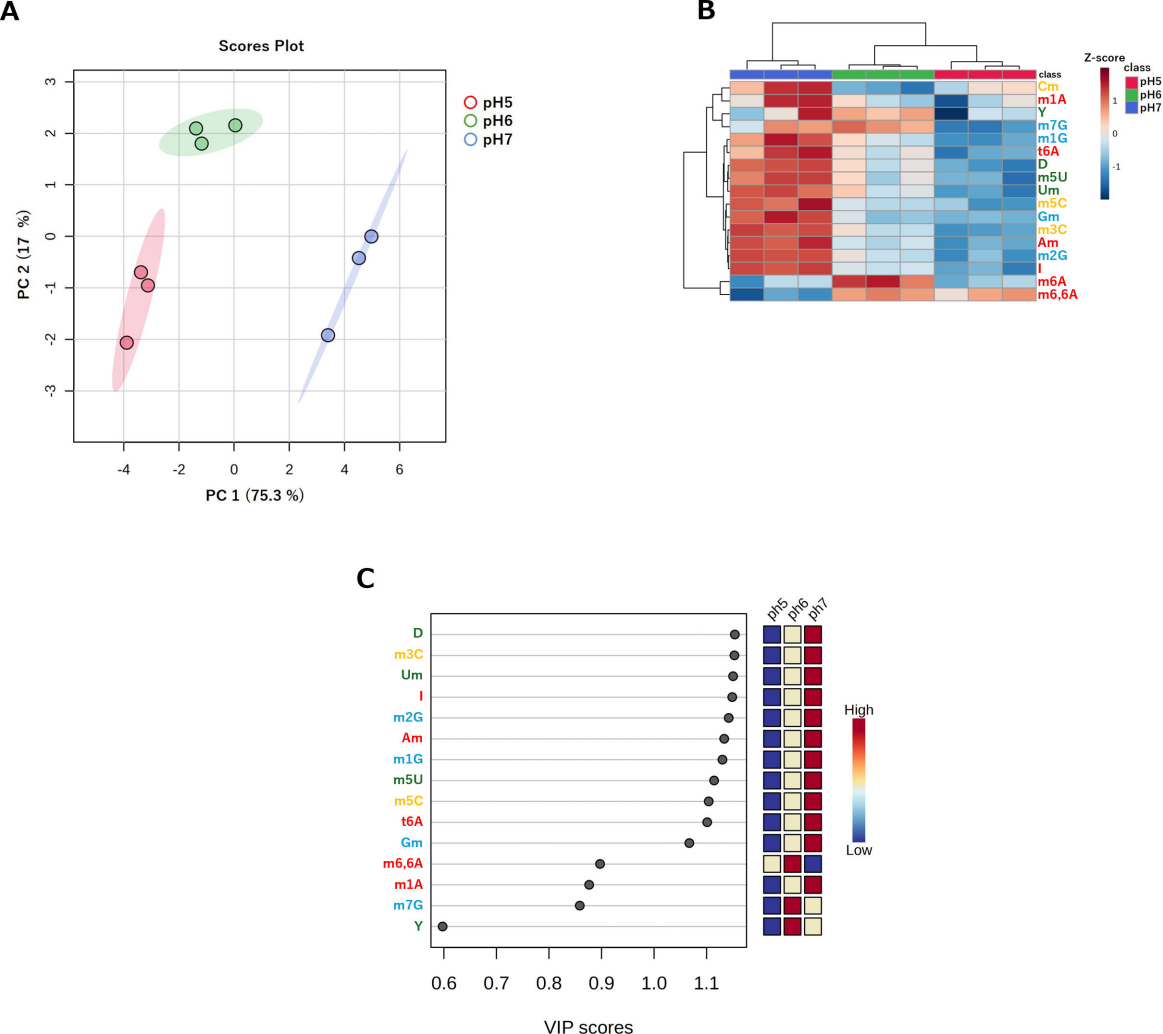
*Ureaplasma* epitranscriptome is dynamically regulated by the environmental pH. All data are from *n* = 3 biological replicates per condition. (**A**) PCA plot of global RNA modification profiles, demonstrating distinct clustering of samples based on pH (pH 5, red; pH 6, green; pH 7, blue). (**B**) Hierarchical clustering heatmap of 17 RNA modifications selected by one-way ANOVA with Tukey’s post-hoc test (FDR-adjusted *P* < 0.05), showing a clear pH-dependent pattern of abundance change. (**C**) VIP score plot from PLS-DA, highlighting the most influential RNA modifications driving the separation between pH groups, with several tRNA modifications showing high scores. To facilitate interpretation, modified RNAs are color-coded and organized by their respective base types: adenosine-based (red text), guanosine-based (blue text), cytidine-based (yellow text), and uridine-based (green text).

To investigate potential post-transcriptional regulatory changes, we analyzed the global RNA modification landscape of these cells. PCA revealed distinct clustering by pH, indicating that the epitranscriptome was dynamically regulated (Fig. 3A). Pairwise volcano plot analyses using pH 6 as the control condition visualized the magnitude and significance of individual modification changes at pH 5 and pH 7, respectively (Fig. S3A and B). A heatmap of individually quantified RNA modifications revealed distinct pH-dependent patterns, with most modifications increasing from pH 5 to pH 7, with the notable exception of N6,N6-dimethyladenosine (m6,6A), which showed the opposite trend (Fig. 3B). This trend was further confirmed by PLS-DA analysis (Fig. S3C). The VIP score plot identified dihydrouridine (D), 3-methylcytidine (m3C), 2′-O-methyluridine (Um), inosine (I), N2-methylguanosine (m2G), and 2′-O-methyladenosine (Am) as the modifications most strongly associated with pH 7. Notably, these modifications span diverse chemical classes, including base modifications on both purines and pyrimidines as well as ribose methylations, suggesting that the increase at pH 7 was not driven by any single modification type or mechanism (Fig. 3C). These data suggest that cellular responses to pH extend to broad remodeling of the epitranscriptome.

### Integrated correlation network analysis identifies co-regulated functional modules

**Fig 4:**
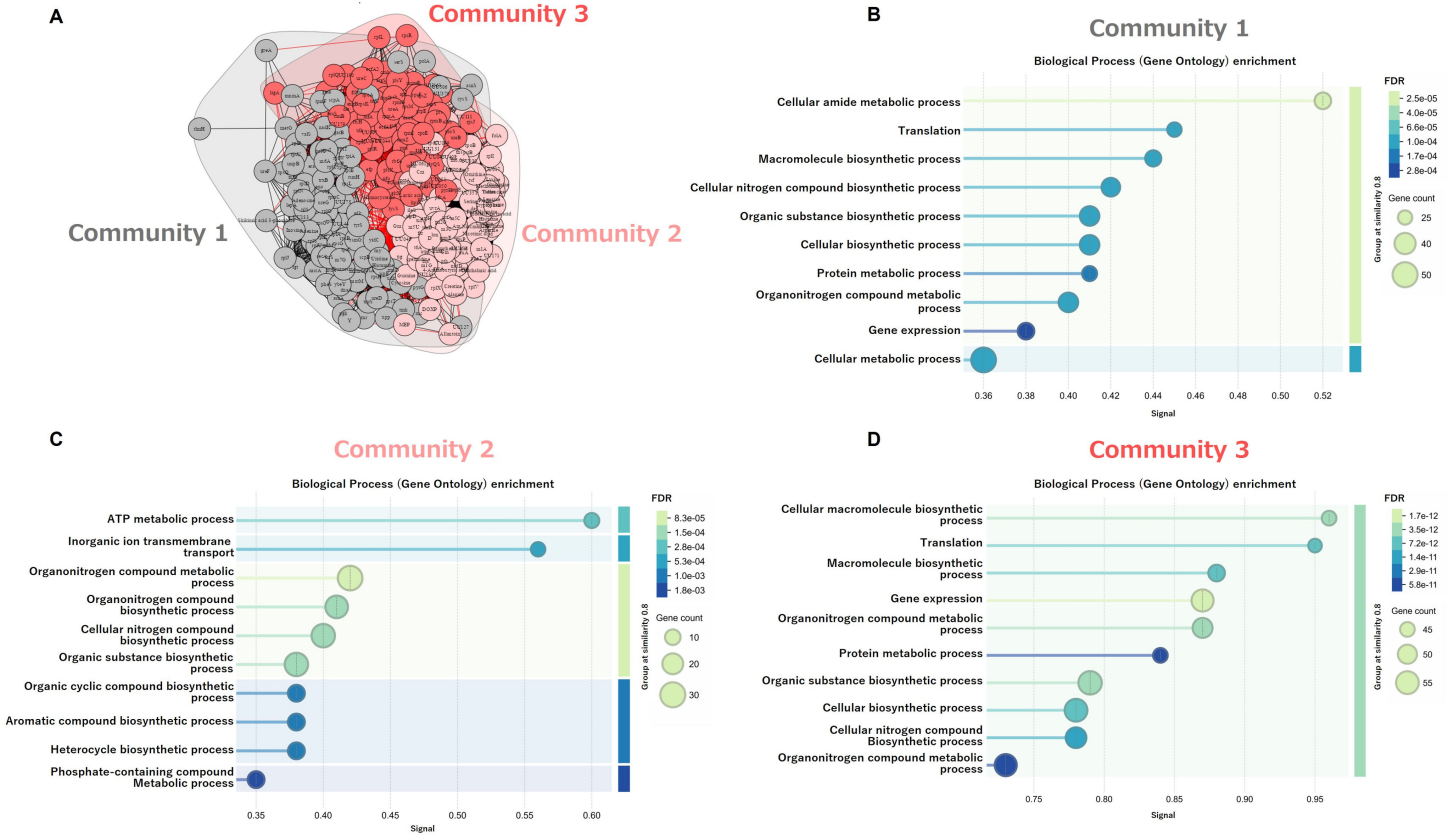
Integrated multi-omics network analysis reveals co-regulated functional modules. The network was constructed using data from three biological replicates per condition. (**A**) Multi-omics correlation network constructed from all measured proteins, metabolites, and RNA modifications. Nodes are colored based on their assignment to one of the three major co-regulated communities: Community 1 (gray), Community 2 (pink), and Community 3 (red). (**B–D**) Gene Ontology (GO) enrichment analysis of the biological processes associated with proteins within each community. (**B**) Community 1 is enriched for translation and metabolic processes. (**C**) Community 2 is enriched in ATP metabolism and transport processes. (**D**) Community 3 is enriched in macromolecule biosynthesis and translation. Bar length represents the significance of enrichment (Signal), dot size represents the number of genes in the category, and dot color represents the FDR.

To move beyond the independent analysis of individual omics layers and understand the system-level coordination of the pH response, we constructed a multi-omics correlation network based on Spearman correlation coefficients between all measured molecules. Crucially, this analysis was performed across all samples regardless of pH conditions, ensuring the resulting structure reflects pH-transcending correlations rather than pH-group-specific features. This analysis revealed that the measured molecules clustered into three major communities of co-varying components (Fig. 4A). We performed enrichment analysis for each community to functionally annotate them. Community 1 was predominantly enriched for terms related to “Cellular amide metabolic process” and “Translation” (Fig. 4B). Community 2 was significantly enriched for “ATP metabolic process” and other energy-related pathways (Fig. 4C). Community 3, like Community 1, was strongly enriched for terms related to “Translation” and biosynthesis, such as “Cellular macromolecule biosynthetic process” (Fig. 4D). This modular organization suggests a universal blueprint composed of a “biosynthesis/translation” system (Communities 1 and 3) and an “energy metabolism” system (Community 2).

Notably, these communities recapitulate and interconnect the pH-responsive functional categories identified independently in our proteomic, metabolomic, and epitranscriptomic analyses (Fig. 1 to 3). The hub network for Community 1 showed a dense cluster of co-regulated ribosomal proteins (e.g., rpsF and rpsM) and translation-related factors (e.g., infA) (Fig. S4A), consistent with the coordinated ribosomal protein upregulation at pH 5 observed in Fig. 1J. The integrated hub network for Community 2 revealed strong positive correlations between energy metabolism proteins (e.g., atpA), transporters (pstB), and amino acids that accumulate at pH 7 (Fig. S4B), linking the proteomic upregulation of ATP synthase subunits (Fig. 1G) with the metabolomic accumulation of amino acids (Fig. 2) into a single co-regulated unit. The hub network for Community three further emphasizes the translational machinery, showing strong correlations among key ribosomal proteins (e.g., rplK, rplA, rplO) and a direct link to the RNA modification m7G (Fig. S4C), suggesting a functional coupling between ribosome abundance and epitranscriptomic regulation that was not apparent from analyzing each omics layer independently. Collectively, these analyses demonstrate that pH-dependent phenotypic changes arise from the differential activation of pre-existing, co-regulated functional modules, rather than from the independent regulation of individual molecules.

## DISCUSSION

The integrated multi-omics data presented here, framed by the finding that *U. parvum* proliferation is optimal at pH 6, allow us to propose a working model of bifurcated adaptation to environmental pH stress. Our multi-omics correlation network analysis suggests that *U. parvum* operates based on modular functional architecture spanning the proteome, metabolome, and epitranscriptome. Within the scope of this study, the most prominent modules were the co-regulated “biosynthesis/translation” system and the “energy metabolism” system. Our data suggest that the differential activation of these pre-existing modules underlies *U. parvum’s* response to pH stress. Under suboptimal acidic pH 5 conditions, cells appear to activate a “biosynthesis/translation” module. The primary evidence for this is a marked increase in ribosomal protein levels. This observation is supported by our metabolome data showing simultaneous accumulation of translation precursors such as nucleosides and the translation-promoting polyamine spermidine. The functional implications of this coordinated upregulation of ribosomes and nucleosides remain unclear, but it may reflect a state in which cells exposed to moderate acid stress maintain a translational capacity. The accumulation of spermidine, in particular, suggests a potential mechanistic link to this interpretation. Polyamines are essential for the structural integrity and function of the translation apparatus (15, 16). As multivalent cationic molecules, they bind to and stabilize the negatively charged rRNA backbone, protecting ribosomes from degradation and ensuring proper conformation. Furthermore, spermidine has been shown to enhance translation accuracy and contribute to acid stress resistance in other species (17). Thus, spermidine accumulation at pH 5 may represent a multifunctional response that simultaneously stabilizes the ribosomal machinery and contributes to acid tolerance.

Conversely, at pH 7, the cells appeared to activate “energy metabolism.” A noteworthy observation was the simultaneous upregulation of F-type ATP synthase subunits and stress response proteins. This co-occurrence suggests that the state of heightened energy metabolism is associated with significant cellular stress. One possible source of this stress is the ammonia production pathway driven by urease, a defining feature of *Ureaplasma*. The *Ureaplasma* urease has been shown to exhibit optimal enzymatic activity at pH 7.5 (18), and its hydrolysis of urea produces large amounts of ammonia, a widely reported cytotoxic substance that disrupts protein structure and function (19, 20). While our proteomics data reflect changes in urease subunit abundance rather than enzymatic activity, the increase in urease subunit abundance at pH 7 is potentially relevant to this mechanism, although direct activity measurements were not performed. Therefore, the marked upregulation of proteostasis machinery (chaperones and proteases) can be interpreted as a response to ammonia toxicity (21). However, it cannot be excluded that the observed stress response partially reflects the direct physicochemical effects of pH shift on protein stability, independent of ammonia production. Regardless of the precise cause, these observations suggest a physiological trade-off at pH 7: while the condition may favor energy production and virulence factor generation, it simultaneously induces a stressful intracellular environment that requires constant management. This interpretation is further supported by the significant accumulation of various amino acids at pH 7. This amino acid accumulation can be understood as a multifaceted consequence of a high-flux and high-stress state. First, it may be a direct consequence of an activated proteostasis network. The degradation of proteins damaged by ammonia toxicity via Lon protease would release a substantial pool of free amino acids for recycling. Second, this enlarged amino acid pool could serve as a ready supply of building blocks for the targeted synthesis of proteins essential for this state, namely the ATP synthase complex and the proteostasis machinery itself. Third, these amino acids may function as compatible solutes, helping to maintain osmotic balance and buffer the intracellular pH against the alkalizing effect of ammonia production, thereby contributing to overall cellular homeostasis under stress (22–25). These possibilities are not mutually exclusive, and the relative contribution of each mechanism remains to be determined.

The expression patterns of ribosomal proteins present a particularly intriguing case in regulation, suggesting that the translational machinery undergoes distinct management under different conditions. The pH 6 state likely represents balanced homeostatic growth requiring a baseline level of translational capacity sufficient for proliferation. In contrast, the substantial upregulation observed at pH 5 exceeds pH 6 levels and, as noted, may reflect translational readiness rather than being associated with active growth. The pH 7 condition differs. Here, ribosomal protein levels were lower than at pH 5 but exceeded the pH 6 baseline. This suggests that the high-stress, high-energy state at pH 7 imposes a specific translational burden distinct from normal growth. This may reflect a shift in translational priorities from the broad protein synthesis required for cell division toward the production of a limited set of proteins essential for survival under these conditions. Maintaining such a high rate of protein turnover, characterized by rapid synthesis and degradation, may require additional layers of translational regulation, and the involvement of RNA modifications represents a plausible mechanism. A novel finding of our study is the dynamic remodeling of the epitranscriptome in response to pH changes. We identified significant changes in several RNA modifications, such as m1G, D, and m5C, which are known to influence the structure and function of tRNA and rRNA (26–28). Of particular note is that analysis of RNA-modifying enzymes across the entire Mollicutes genome predicted a conserved set of modifications, including m7G, in *U. parvum* (29). Our experimental data directly corroborated the presence of these predicted modifications with biochemical evidence and demonstrated that their levels are dynamically regulated in response to pH. Despite extensive genome reduction in Mollicutes, the retention of these specific modification enzymes suggests their functional importance. Correlation network analysis notably shows that RsmG, a putative m7G methyltransferase, resides within the hub network of Community three alongside ribosomal proteins, yet exhibits a negative correlation with m7G modification levels. The biological significance of this inverse correlation remains unclear and warrants further investigation. However, it is entirely plausible that the relationship between enzyme expression levels and RNA modification levels is not a simple correlation. Additional factors, such as cofactor availability, RNA metabolic turnover rates, and post-translational regulation of enzyme activity may also contribute to this relationship. Furthermore, RNA modifications influence translation speed and accuracy. These modifications provide a mechanism for fine-tuning protein synthesis without altering gene transcription. In organisms lacking many transcriptional regulators, such as *Ureaplasma*, post-transcriptional control may become the primary means of regulating protein expression. For example, specific modifications have been reported to promote selective translation of stress-related transcripts through codon-biased mechanisms in other organisms (30–32), suggesting that similar regulatory principles may operate in *Ureaplasma* under pH stress.

This reliance on post-transcriptional and translational regulation is likely intertwined with another fundamental feature of the *Ureaplasma* genome: its high AT content (33). An AT-rich genome transcribes AU-rich mRNAs, which can present challenges for translational fidelity and efficiency because of codon bias and altered RNA stability (34). The pH-dependent modulation of the translational machinery, as evidenced by large-scale changes in ribosomal protein abundance and dynamic remodeling of tRNA and rRNA modifications, may represent a system for the effective management of AU-rich transcripts. This could ensure that the correct proteins are synthesized at the appropriate levels for survival and growth.

Furthermore, high AT content can be viewed as an energy conservation strategy. As adenine–thymine (A–T) pairs are joined by two hydrogen bonds, compared with the three bonds in guanine–cytosine (G–C) pairs, less energy is required to unwind the DNA helix during replication and transcription (35). This genomic feature aligns with the efficient metabolic switching observed in this study, underscoring a deeply integrated evolutionary strategy for survival in resource-limited organisms.

This study emphasizes the use of integrated multi-omics analysis as a valuable strategy for understanding the biology of minimal organisms. Although the small genome of *Ureaplasma* may suggest simplicity, our data suggest the existence of a multilayered regulatory network spanning the proteome, metabolome, and epitranscriptome. These layers influence each other, and only by measuring them simultaneously can we potentially provide deep insights into the mechanisms of cellular adaptation. This approach effectively circumvents the limitations of organisms that are difficult to study using conventional genetic methods or transcriptome analysis due to the scarcity of known regulatory factors. The regulatory networks identified here suggest that minimal organisms may have developed post-transcriptional and metabolic control mechanisms during evolution to compensate for their reduced genomic toolkit. Although this study provides a system-level framework for understanding *Ureaplasma* pH adaptation, it has certain limitations. First, our experiments were conducted under controlled *in vitro* batch culture conditions. The *in vivo* environment of the human urogenital tract is significantly more complex, involving host-microbe interactions, fluctuating nutrient availability, and the presence of a polymicrobial community. Future studies using co-culture models or direct analysis of clinical specimens are necessary to assess whether our proposed model accurately reflects *Ureaplasma* behavior within the host. Furthermore, since this study was conducted using a single cell line, it will also be necessary to examine *Ureaplasma* with different genomic backgrounds. Second, our multi-omics analysis provided a static snapshot of cells at a single 16-h sampling point. The dynamics of adaptation over time were not fully captured. Time-course experiments that sample cells at various points following a pH shift would provide valuable insights into the temporal orchestration of adaptive responses. Third, transcriptomic data were not included in this study. Our central hypothesis is that post-transcriptional regulation is important in this minimal organism. However, the absence of mRNA-level data prevents us from definitively decoupling the effects of translational control from potential transcriptional changes. Integrating transcriptomics in future studies would allow for a more complete understanding of the regulatory cascade. Fourth, our metabolomic analysis used a semi-untargeted approach. Although this method covers a broad range of key compounds, it is not exhaustive, and other unquantified metabolites may contribute to the adaptive response. Finally, while correlation network analysis identified strong correlations between molecules, it did not establish causality. For example, while the data support the hypothesis of “translational regulation” mediated by RNA modifications, direct functional validation—such as genetic manipulation of modification enzymes, which remains challenging in *Ureaplasma*—is necessary to establish causality. These limitations highlight key directions for future research building on the framework presented in this study.

### Conclusion

This integrated multi-omics analysis provides compelling evidence for a molecular switch in *Ureaplasma* that governs its response to environmental pH. Our data indicate that *Ureaplasma* shifts from a state of balanced proliferation at its optimal growth pH of 6 to a state characterized by an upregulated translational apparatus under acidic stress and a high-stress, “bioenergetic activation” state under alkaline conditions. This is achieved through the differential activation of universal, co-regulated functional modules, a process fueled by distinct metabolic shifts and likely fine-tuned by dynamic RNA modifications.

This study highlights how a minimal-genome organism can achieve remarkable adaptive plasticity, not through complex transcriptional networks, but through sophisticated post-transcriptional and translational control. Using *Ureaplasma* as a model, this study provides a new framework for understanding the physiology and pathogenicity of this organism and offers a powerful methodological approach for understanding the fundamental principles of life at its minimal edge.

## MATERIALS AND METHODS

### Bacterial strain and culture conditions

The clinical isolate of *U. parvum*, OMC-P162 (36), was cultured at 37°C in defined UMCHs medium (37) adjusted to an initial pH of 5, 6, or 7. For multi-omics analysis, cultures were inoculated at an initial density of 1.7 × 10³ CFU/mL and incubated at 37°C for 16 h, from 17:00 to 09:00 the following day, after which the cells were harvested. After incubation, the cultures were centrifuged at 16,000 × g for 20 min, and the resulting bacterial pellets were stored at −80 °C until further use. Viable cell counts and medium pH were measured at the 16-h sampling point under the same initial pH conditions, as shown in Fig. S1A. For the additional time-course turbidity and pH measurements shown in Fig. S1B and C, cultures were prepared in scaled-up 20-mL volumes under each initial pH condition to enable serial monitoring of culture dynamics. Cultures were initiated with the same viable inoculum of 1.7 × 10³ CFU/mL, corresponding to 3.4 × 10⁴ CFU per culture bottle. Subsequent changes in culture density were monitored as relative culture turbidity, and the pH of the culture medium was measured in parallel. Because the culture volume differed from that used for the multi-omics sampling experiment, these time-course data were used as supporting information to evaluate culture dynamics under the three initial pH conditions.

### Sample preparation for proteomics

Cell pellets were resuspended in radioimmunoprecipitation assay buffer (Fujifilm Wako Pure Chemical Corporation, Osaka, Japan) and lysed on ice. Protein concentration was measured using a BCA protein assay kit (Thermo Fisher Scientific, Waltham, MA, USA). Proteins were reduced with 10 mM TCEP (Thermo Fisher Scientific) and alkylated with 18 mM iodoacetamide (Thermo Fisher Scientific). Before digestion, proteins were pelleted using methanol/chloroform precipitation and digested overnight at 37°C with trypsin (Thermo Fisher Scientific).

### LC-MS/MS for proteomics

Peptide samples were analyzed using a Vanquish Neo UHPLC system coupled to an Orbitrap Astral mass spectrometer (Thermo Fisher Scientific). Peptides were separated on an EASY-Spray LC column (Thermo Fisher Scientific) maintained at 55°C. The mobile phases consisted of 0.1% formic acid in water (mobile phase A) and 80% acetonitrile with 0.1% formic acid (mobile phase B). The gradient was initiated at 1.3 µL/min with 4% B for 0.5 min. The flow rate was then changed to 0.8 µL/min, and the gradient was increased to 8% B over 0.4 min, followed by an increase to 22.5% B over 13 min, and then to 35% B over 6.9 min. The flow rate was changed to 2 µL/min, and the gradient was increased to 55% B over 0.4 min, followed by a final increase to 99% B over 1.3 min, before column washing and re-equilibration. The mass spectrometer was operated in data-independent acquisition (DIA) mode. The method comprised a repeating cycle of one full MS scan, followed by DIA scans within a 23-min run. The full MS scan was acquired in the Orbitrap analyzer at a resolution of 240,000 over a scan range of 380–980 m/z, with a normalized automatic gain control (AGC) target of 500% and a maximum injection time of 5 ms. Following the full scan, 300 DIA scans were acquired using an Astral Analyzer. The HCD collision energy was set to a normalized value of 25. For the Astral detector, the scan range was 150–2,000 m/z, the normalized AGC target was 500%, and the maximum injection time was 3 ms. Raw data files were processed using DIA-NN (version 2.2.0) and searched against the *U. parvum* FASTA database (Taxonomy ID: 38504) downloaded from UniProt.

### Metabolite extraction

Cell pellets were subjected to metabolite extraction by adding 400 µL of ice-cold methanol containing internal standards (1 µM MES and 1 µM methionine sulfone), followed by the addition of 400 µL chloroform. The mixture was vortexed for 1 min, after which 320 µL of water was added, and the sample was vortexed again. The sample was incubated on ice for 5 min and centrifuged at 16,000 *× g* for 5 min. The upper aqueous phase was collected, evaporated to dryness using a SpeedVac concentrator (Thermo Fisher Scientific), and reconstituted in 20-µL ultrapure water for mass spectrometric analysis.

### LC-MS/MS for metabolomics

Metabolomic analysis was conducted using a semi-untargeted method on a Nexera X3 UHPLC system coupled to an LCMS-8060NX triple-quadrupole mass spectrometer (Shimadzu Corporation, Kyoto, Japan). The analytical method was based on the “LC/MS/MS Method Package for Primary Metabolites ver.3” (Shimadzu Corporation), which was customized by incorporating an in-house library of additional metabolites. Chromatographic separation was performed using a Discovery HS F5-3 column (2.1 mm I.D. × 150 mm, 3 µm; Sigma-Aldrich, Saint Louis, MO, USA). Metabolites were identified and quantified using a scheduled multiple reaction monitoring (MRM) method. Detailed MRM transitions and other analytical conditions for the custom-added metabolites are provided in the Supplementary File (Table S1). Raw data files were processed using Traverse MS software (Shimadzu Corporation) for peak alignment and integration to calculate peak areas.

### RNA extraction and digestion for modification analysis

Total RNA was extracted from the cell pellets using TRIzol reagent (Invitrogen, Carlsbad, CA, USA) according to the manufacturer protocol. Total RNA was digested with 0.5 units of nuclease P1 (Fujifilm Wako Pure Chemical Corporation) at 45°C for 2 h, followed by incubation with bacterial alkaline phosphatase (Takara Bio, San Jose, CA, USA) at 37°C for 2 h to generate the nucleosides. After deproteinization with chloroform, the aqueous phase containing the nucleotides was collected, evaporated to dryness, reconstituted in ultrapure water, and analyzed using mass spectrometry. An internal standard, ¹⁵N₅-deoxyguanosine (dG15N5), was added to each sample before digestion.

### LC-MS/MS for RNA modification analysis

Nucleosides were analyzed using a Nexera X3 UHPLC system coupled to an LCMS-8060NX triple quadrupole mass spectrometer (Shimadzu Corporation). Chromatographic separation was achieved using an ACQUITY UPLC CSH C18 Column (130 Å, 1.7 µm, 2.1 mm × 100 mm; Waters Corporation, Milford, MA, USA). The mobile phases consisted of mobile phase A (5 mM ammonium acetate in water) and mobile phase B (5 mM ammonium acetate in methanol). A multi-step gradient was applied at a flow rate of 0.2 mL/min as follows: a linear gradient to 32% B over 6 min, a rapid increase to 99% B from 6.0 to 6.5 min, an isocratic hold at 99% B until 10.5 min, a rapid return to 1% B from 10.5 to 10.6 min, and re-equilibration at 1% B until 15.0 min. The detailed MRM parameters for each nucleoside are provided in a supplementary file (e.g., Table S2). Raw data were processed using Traverse MS software (Shimadzu Corporation) for peak alignment and data integration. To determine the relative abundance of each modification, we normalized the peak area of each modified nucleoside to that of the internal standard (dG15N5). This value was normalized to the peak area of the corresponding unmodified canonical nucleoside.

### Statistical and bioinformatic analyses

The quantitative proteomics data matrix from DIA-NN was processed using Perseus software for data filtering, transformation, and imputation. Proteins with more than 50% missing values in any experimental group were excluded. The remaining intensity values were log2-transformed, and the missing values were imputed based on a normal (Gaussian) distribution. Volcano plots were generated within Perseus to visualize differentially abundant proteins, which were determined using a two-sided Student’s *t*-test with the following parameters: 250 randomizations, a permutation-based FDR of 0.05, and an S0 of 0.1. PCA of processed proteomics data was performed using MetaboAnalyst 6.0. For metabolite profiling data and RNA modification data, both PCA and PLS-DA were performed using MetaboAnalyst 6.0. For metabolite profiling data, sum of normalization, log transformation, and auto-scaling were performed using peak area values obtained from mass spectrometry. For RNA modification data, log transformation and auto-scaling were performed using peak areas normalized with the internal standard dG15N5 as input. PLS-DA was used as a supervised method to maximize group separation and identify discriminating features. VIP scores were calculated from the PLS-DA models to rank the degree to which each variable contributed to group discrimination. The VIP score represents the weighted sum of squares of the PLS loadings considering the explanatory variables in each dimension. Variables with VIP scores exceeding 1.0 were considered significant contributors. For heatmap generation, one-way ANOVA and Tukey’s post-hoc test were performed within MetaboAnalyst. Only variables with FDR-adjusted *P* values < 0.05 were included in the heatmaps (metabolite analysis: 38 metabolites; RNA modification analysis: 17 modifications). Hierarchical clustering was performed using Euclidean distance and Ward’s agglomerative method. For network integration, all multi-omics data were filtered for low-variance features and normalized using Z-score transformations. All analyses were performed in R (version 4.3.3). To integrate the data sets, we constructed an inter-omics correlation network by calculating Spearman’s rank correlation coefficients (*ρ*) for all pairwise features between the different omics layers. A network was built by retaining only significant correlations, where |*ρ*| > 0.9 as edges. The Louvain algorithm was used to detect densely connected modules (communities) within the network, and key nodes (hubs) were identified by calculating their degree and betweenness centrality. The final network was visualized using the igraph R package. Functional enrichment analysis was conducted using the STRING database (version 12.0) (https://string-db.org/).

## Data Availability

The metabolomics data generated in this study have been deposited in the DDBJ MetaboBank under accession number MTBKS280. All other data supporting the findings of this study are available from the corresponding author upon reasonable request.

## SUPPLEMENTAL MATERIAL

Figure S1 spectrum.03463-25-s0001.tif Viable cell counts and supporting time-course turbidity/pH measurements of *U. parvum* cultures under different initial pH conditions.

Figure S2 spectrum.03463-25-s0002.tif (A) Volcano plot showing differentially accumulated metabolites at pH 5 relative to pH 6. (B) Volcano plot showing differentially accumulated metabolites at pH 7 relative to pH 6. Solid lines indicate the significance thresholds of fold change (greater than or equal to) 1.2 and *P* value < 0.05. (C) PLS-DA plot among pH groups in the metabolome.

Figure S3 spectrum.03463-25-s0003.tif (A) Volcano plot showing differentially accumulated metabolites at pH 5 relative to pH 6. (B) Volcano plot showing differentially accumulated RNA modifications at pH 7 relative to pH 6. Solid lines indicate the significance thresholds of fold change (greater than or equal to) 1.2 and *P* value < 0.05. (C) PLS-DA plot confirming the distinct separation of epitranscriptomic profiles by pH.

Figure S4 spectrum.03463-25-s0004.tif Hub network analysis highlights the key molecular interactions within each functional community.

Supplemental legends spectrum.03463-25-s0005.docx Legends for Figures S1 to S5.

Tables S1 to S5 spectrum.03463-25-s0006.xlsx Measurement conditions for mass spectrometry and omics data sets.

ASM does not own the copyrights to Supplemental Material that may be linked to, or accessed through, an article. The authors have granted ASM a non-exclusive, world-wide license to publish the Supplemental Material files. Please contact the corresponding author directly for reuse.
