# Supplementary material for: Integrated multi-omics analysis reveals a pH-driven metabolic and translational switch in Ureaplasma parvum
Source: Microbiol Spectr. 2026 Jul 10;14(8):e03463-25. doi: 10.1128/spectrum.03463-25 (PMC13436400; doi:10.1128/spectrum.03463-25)
Supplement: Supplementary file 1 [file spectrum.03463-25-s0005.docx]

**Supplementary Figure 1. Viable cell counts and supporting time-course turbidity/pH measurements of U. parvum cultures under different initial pH conditions.** (A) Viable cell counts and medium pH at the 16-h sampling point used for multi-omics analysis. Cultures were initiated at pH 5, 6, or 7 and harvested after 16 h of incubation. (B) Time-course changes in relative culture turbidity measured in scaled-up 20-mL cultures under the three initial pH conditions. (C) Time-course changes in medium pH measured in parallel with the turbidity measurements. Cultures in B and C were initiated with 1.7 × 10³ CFU/mL, corresponding to 3.4 × 10⁴ CFU per culture bottle. At 48 h, the final pH values were 6.57 ± 0.01, 7.45 ± 0.02, and 8.27 ± 0.02 for cultures initiated at pH 5.0, 6.0, and 7.0, respectively.

**Supplementary Figure 2.**  (A) Volcano plot showing differentially accumulated metabolites at pH 5 relative to pH 6. (B) Volcano plot showing differentially accumulated metabolites at pH 7 relative to pH 6. Solid lines indicate the significance thresholds of fold change ≥ 1.2 and p-value < 0.05. (C) PLS-DA plot among pH groups in the metabolome.

**Supplementary Figure 3.**  (A) Volcano plot showing differentially accumulated metabolites at pH 5 relative to pH 6. (B) Volcano plot showing differentially accumulated RNA modifications at pH 7 relative to pH 6. Solid lines indicate the significance thresholds of fold change ≥ 1.2 and p-value < 0.05. (C) PLS-DA plot confirming the distinct separation of epitranscriptomic profiles by pH.

**Supplementary Figure 4. Hub network analysis highlights the key molecular interactions within each functional community.** (A) Hub network for Community 1, showing a densely interconnected module of ribosomal proteins (e.g., rpsF, rpsM) and translation initiation factors (e.g., infA). (B) Integrated hub network for Community 2, revealing strong positive correlations (red lines) between energy metabolism proteins (e.g., atpA, green nodes), transporters, and key amino acids (e.g., Proline, Arginine, beige nodes) that accumulate at pH 7. (C) Hub network for Community 3, illustrating strong correlations among major ribosomal proteins (e.g., rplK, rplA, rplO) and a direct link to the RNA modification m7G. In all networks, the node size is proportional to its degree of connectivity, and the thickness of the connecting line (edge) represents the strength of the correlation.

**Supplementary Table 1.** Analytical conditions for additional metabolites incorporated in the in-house extended Method Package v3 used for metabolomic analysis.

**Supplementary Table 2.** List of analytical conditions employed for RNA modification profiling.

**Supplementary Table 3.** Comprehensive proteomic dataset of Ureaplasma generated in this study.

**Supplementary Table 4.** Comprehensive metabolomic dataset of Ureaplasma.

**Supplementary Table 5.** Comprehensive list of RNA modification profiles identified in Ureaplasma.
